# Xanthelasma‐Like Reaction Following Poly‐L‐Lactic Acid Intradermal Injection Managed With Fractional Non‐Ablative Lasers and Stem Cell‐Derived Conditioned Media: A Case Report

**DOI:** 10.1111/jocd.70739

**Published:** 2026-02-16

**Authors:** Olivia Wibisono, Martha Fang, Kyu‐Ho Yi, Irwan Junawanto

**Affiliations:** ^1^ Flora House of Beauty Clinic Jakarta Indonesia; ^2^ Martha Fang Skin Clinic Bali Indonesia; ^3^ Division of Anatomy and Developmental Biology, Department of Oral Biology Yonsei University College of Dentistry Seoul Republic of Korea; ^4^ ERHA Dermatology Clinic Jakarta Indonesia

**Keywords:** 1450‐nm diode laser, 1550‐nm erbium glass, collagen stimulator, non‐ablative fractional laser, poly‐D,L‐lactic acid, poly‐L‐lactic acid, stem cell‐derived conditioned media, xanthelasma‐like reaction

## Abstract

**Background:**

Xanthelasma‐like periocular plaques are rare adverse reactions following injectable collagen stimulators and may be challenging to manage in cosmetically sensitive areas.

**Aims:**

To describe a rare xanthelasma‐like reaction after polymer‐based collagen stimulator (poly‐L‐lactic acid or poly‐D,L‐lactic acid) intradermal injection and its management using fractional non‐ablative lasers and stem cell‐derived conditioned media.

**Patients/Methods:**

A 49‐year‐old woman with Fitzpatrick skin type IV developed bilateral infraorbital yellowish plaques (~1 × 2 cm) approximately two weeks after a collagen stimulator injection and presented four months later with persistent lesions, after showing minimal response to a prior 577‐nm yellow laser treatment performed at another clinic. Our first treatment approach consisted of a fractional 1550‐nm erbium glass laser combined with intralesional 0.9% sodium chloride and a short course of oral corticosteroids. A subsequent session incorporated combined fractional 1550‐nm erbium glass and 1450‐nm diode lasers, followed by intradermal stem cell‐derived conditioned media.

**Results:**

Partial improvement was observed after the initial treatment. Further improvement was achieved following combination laser therapy with adjunctive stem cell‐derived conditioned media, with marked lesion flattening and reduced erythema within two months and sustained improvement at four‐month follow‐up.

**Conclusions:**

This case highlights a rare xanthelasma‐like reaction following polymer‐based collagen stimulator injection and demonstrates that a staged, non‐ablative fractional laser approach with adjunctive stem cell‐derived conditioned media may be an effective and minimally invasive management option.

## 1 Introduction

Poly‐L‐lactic acid (PLLA) and poly‐D,L‐lactic acid (PDLLA) are biodegradable polymers widely used as collagen stimulators for facial rejuvenation and skin texture improvement [1, 2]. Although these agents are generally safe, delayed inflammatory or lipid‐laden reactions may occur, occasionally presenting as xanthelasma‐like plaques [3, 4, 5]. Recognition and management of these polymer‐associated reactions are essential to avoid overtreatment or unnecessary excision.

## 2 Case Presentation

A 49‐year‐old woman presented with firm, slightly elevated yellowish plaques (~1 × 2 cm) beneath both lower eyelids, which developed approximately two weeks after a collagen stimulator injection performed with a blunt cannula and persisted for four months before presentation. The patient had Fitzpatrick skin phototype IV. She reported one prior session of a yellow (577‐nm) laser at another clinic, with minimal improvement.

Our first treatment approach included a non‐ablative fractional 1550‐nm erbium glass laser (pulse energy 5 mJ, density 256 pixels/cm^2^, three passes), intralesional 0.9% sodium chloride (total volume 0.3 mL for both under eye‐lesions), and oral methylprednisolone 8 mg twice daily for three days. The procedure was well tolerated, and only mild transient erythema was noted immediately post‐treatment.

**FIGURE 1:**
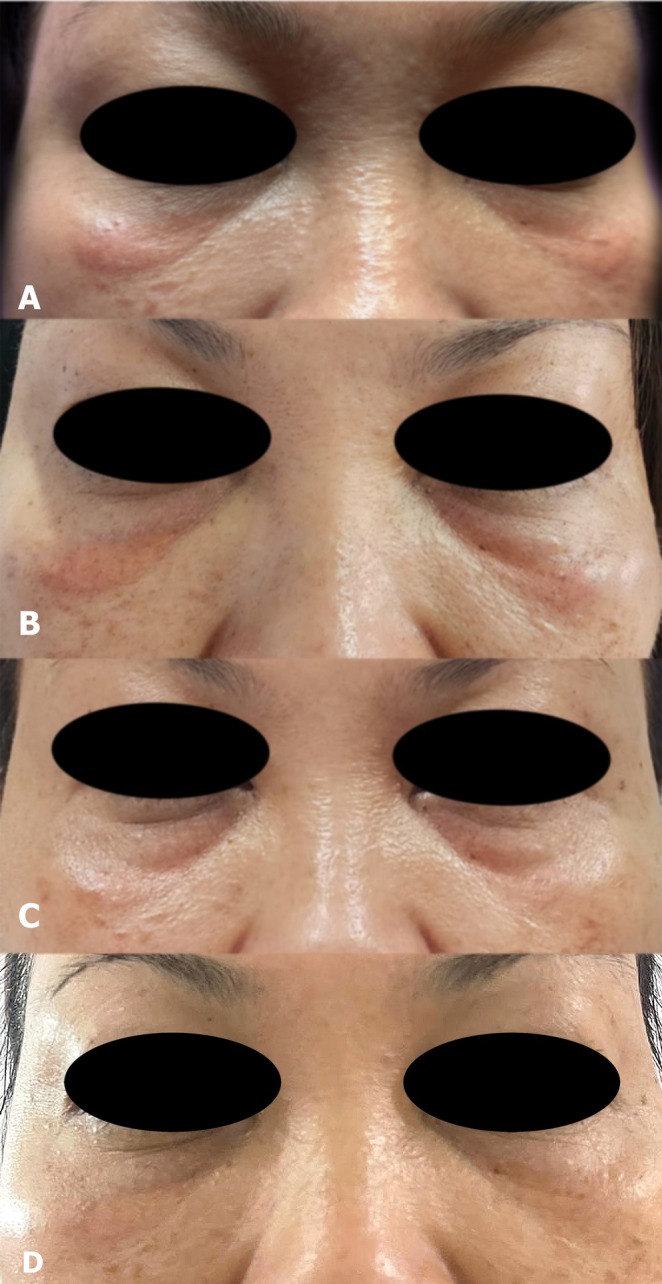
Sequential clinical improvement of bilateral infraorbital xanthelasma‐like lesions. (A) Baseline: Elongated yellowish plaques (~1 × 2 cm) with mild erythema and induration beneath both lower eyelids, four months after collagen stimulator injection. (B) One‐month follow‐up: Partial flattening and reduced erythema following fractional 1550‐nm erbium glass laser treatment, intralesional saline, and a short course of oral corticosteroids. (C) Two‐month follow‐up: Near‐complete resolution after combined fractional 1550‐nm erbium glass and 1450‐nm diode laser treatment with adjunctive intradermal stem cell‐derived conditioned media. (D) Four‐month follow‐up: Sustained clinical improvement with no recurrence of infraorbital plaques.

At one month, partial improvement was noted with flattening and reduced erythema. A second‐stage treatment combined a non‐ablative fractional 1550‐nm erbium glass laser using the same parameters as in the initial session and a 1450‐nm diode laser (spot size 6 mm, fluence 4.5 J/cm^2^, pulse duration 20 ms, frequency 10 Hz), followed by intradermal injection of umbilical cord‐derived mesenchymal stem cell (UC‐MSC) conditioned media (0.2 mL per under‐eye lesion). The UC‐MSC‐derived conditioned media utilized in this study was produced by PT Bifarma Adiluhung, Jakarta, Indonesia, an accredited good manufacturing practices (GMP) facility certified by the Indonesian Food and Drug Authority (certification number: PWS.01.04.1.3.333.09.21‐0082). No adverse effects occurred. At two months, the lesion had nearly resolved, showing smoother texture and normalized pigmentation (Figure 1). At four months, improvement was sustained without recurrence (Figure 1D).

**FIGURE 2:**
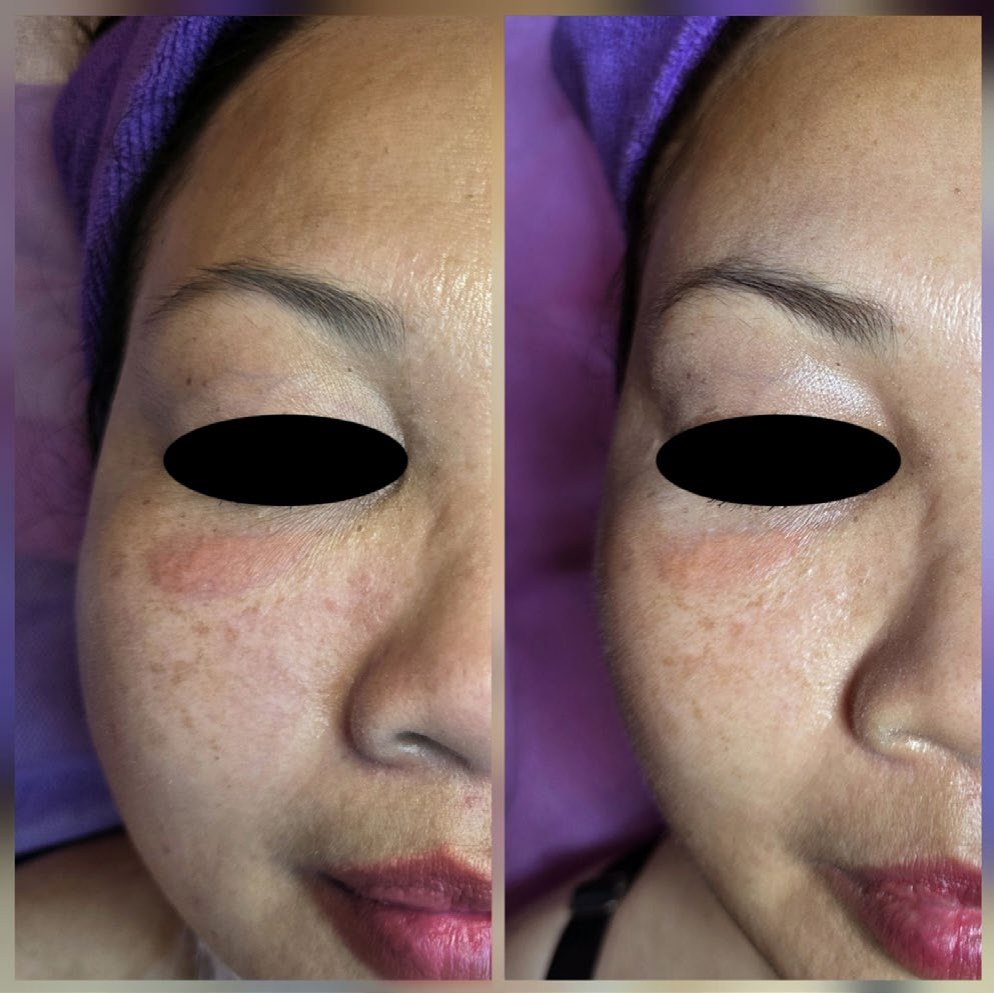
Side‐by‐side comparison of the right infraorbital lesion at baseline (left) and one‐month follow‐up (right), showing visible reduction in erythema, induration, and plaque thickness.

Side‐by‐side photographs of the right infraorbital region demonstrate improvement between the first visit and one‐month follow‐up, with a visible reduction in erythema and plaque thickness (Figure 2).

## 3 Discussion

Xanthelasma‐like reactions following injectable fillers are uncommon and have been reported most frequently after hyaluronic acid and calcium hydroxylapatite injections, with only limited reports involving polymer‐based collagen stimulators such as poly‐L‐lactic acid (PLLA) or poly‐D,L‐lactic acid (PDLLA) [6]. Previously published cases typically describe delayed‐onset reactions, occurring several months to over one year after injection, often attributed to chronic inflammatory or foreign‐body responses. In contrast, the present case demonstrated an earlier onset, with lesion development approximately two weeks after injection, suggesting a potentially different pathophysiologic mechanism.

Although the patient could not confirm the exact commercial product used, she reported receiving a collagen‐stimulating injectable filler. The clinical presentation, anatomic distribution, and temporal association were consistent with reactions described following intradermal PLLA or PDLLA injections. Polymer‐based fillers induce neocollagenesis through a controlled inflammatory response; however, in certain individuals, exaggerated macrophage activation and altered local lipid metabolism may result in lipid‐laden, xanthelasma‐like plaques. The relatively early onset observed in this case may reflect superficial intradermal placement, the thin periocular dermis, or an individual immune‐mediated response rather than a delayed granulomatous foreign‐body reaction.

## 4 Differential Diagnosis

The differential diagnosis for yellowish periocular plaques includes true xanthelasma palpebrarum, granulomatous filler reaction, necrobiotic xanthogranuloma, and sarcoidosis. True xanthelasma typically presents as soft, yellow plaques and is often associated with dyslipidemia; however, the close temporal association with filler injection and accompanying inflammatory features made this diagnosis less likely. Granulomatous filler reactions usually present as firm nodules and most commonly occur months to years after injection. Necrobiotic xanthogranuloma and sarcoidosis were considered; however, the absence of systemic symptoms, lack of lesion progression, and favorable response to conservative therapy rendered these diagnoses less probable.

Histopathologic confirmation was not pursued because of the cosmetically sensitive periocular location and the patient's preference to avoid invasive diagnostic procedures. The diagnosis of a xanthelasma‐like reaction was therefore made based on clinical morphology, temporal association with filler injection, and response to treatment.

## 5 Treatment Rationale and Clinical Course

Management of polymer‐related adverse reactions remains challenging, and no standardized treatment algorithm exists. In this case, a staged, minimally invasive approach was selected. A fractional 1550‐nm erbium glass laser was chosen initially for its ability to induce controlled dermal remodeling and modulate inflammatory processes while preserving the epidermis [7, 8]. As demonstrated in Figures 1A and 1B, noticeable improvement was observed after the initial treatment session consisting of fractional 1550‐nm erbium glass laser, intralesional saline, and a short course of oral corticosteroids.

Incremental improvement—from partial flattening at one month to near‐complete resolution at two months (Figure 1C)—suggests that the subsequent addition of a 1450‐nm diode laser and intradermal stem cell‐derived conditioned media provided synergistic benefit. The additional laser session may have contributed to further dermal remodeling and plaque softening. Stem cell‐derived conditioned media contain multiple bioactive factors with reported immunomodulatory, anti‐inflammatory, and pro‐regenerative effects on the skin microenvironment, which may support resolution of persistent inflammatory or foreign‐body‐related reactions and promote tissue recovery [9, 10]. These treatment decisions were empirical and reflective of real‐world clinical practice rather than an attempt to isolate the effect of a single modality.

Spontaneous resolution of xanthelasma‐like reactions following filler injections has been inconsistently reported in the literature, and lesions may persist for months without intervention, particularly when associated with inflammatory or foreign‐body responses. In this context, a conservative, nonsurgical management strategy was favored.

## 6 Limitations

This report has several limitations. The exact filler product and injected concentration could not be confirmed, histopathologic confirmation was not obtained, and the use of multimodal therapy precludes attribution of clinical improvement to a single intervention. Nevertheless, the favorable outcome highlights a practical and conservative management strategy for a rare adverse reaction occurring in a cosmetically sensitive area.

## 7 Conclusion

This case illustrates that polymer‐based collagen stimulators such as poly‐L‐lactic acid and poly‐D,L‐lactic acid, although widely used, may rarely induce xanthelasma‐like periocular reactions. A staged, minimally invasive management approach incorporating fractional non‐ablative laser therapy was associated with progressive lesion flattening and sustained clinical improvement. Adjunctive use of stem cell‐derived conditioned media may further support tissue recovery [9, 10]. Awareness of this uncommon adverse event and its potential management strategies is important for clinicians performing aesthetic injectable procedures.

## Funding

Partial financial support for the article processing charge was provided by Regenic.

## Ethics Statement

Written informed consent was obtained from the patient for the publication of clinical details and images. Institutional review board approval was not required for this single case report.

## Conflicts of Interest

Regenic is affiliated with PT Bifarma Adiluhung, the manufacturer of the conditioned media used in this study. Regenic provided partial financial support for the article processing charge. The sponsor had no role in study design, data collection, analysis, interpretation, or manuscript preparation.

## Data Availability

The data that support the findings of this study are available from the corresponding author upon reasonable request.
